# The BaeS/BaeR two-component system enhances *Vibrio cholerae* intestinal colonization by upregulating *salX*

**DOI:** 10.3389/fcimb.2026.1886586

**Published:** 2026-07-23

**Authors:** Qiushi Wang, Xiaoya Guo, Yongquan Shi, Linyuan Xue, Dongming Xing, Ting Ye

**Affiliations:** 1 Molecular Medicine Innovation Center, Cancer Institute of The Affiliated Hospital of Qingdao University, Qingdao University, Qingdao, China; 2 Department of Clinical Laboratory Center, Shandong Second Provincial General Hospital, Jinan, China

**Keywords:** ABC transporter, BaeS/BaeR, intestinal colonization, L-arginine, *Vibrio cholerae*

## Abstract

**Introduction:**

*Vibrio cholerae* relies on two-component signal transduction systems to adapt to the host intestinal microenvironment. This study investigated the role and regulatory mechanism of the BaeS/BaeR two-component system in intestinal colonization and antimicrobial peptide resistance.

**Methods:**

A *baeS/baeR* deletion mutant and complemented strain were constructed in the O1 El Tor clinical isolate EL2382. Intestinal colonization, Caco-2 cell adhesion, histopathological analysis, transcriptomic profiling, qRT-PCR, western blotting, electrophoretic mobility shift assays, chromatin immunoprecipitation-qPCR, and antimicrobial peptide resistance assays were performed.

**Results:**

Expression of baeS and baeR was markedly induced during intestinal colonization and adhesion to Caco-2 cells. The *ΔbaeS/baeR* mutant exhibited significantly impaired bacterial adhesion and *in vivo* colonization, accompanied by reduced histopathological scores, and these phenotypes were restored by genetic complementation. Transcriptomic analysis identified *salX* (VC2553), which encodes an ABC transporter involved in antimicrobial peptide homeostasis, as a key downstream target of BaeS/BaeR. BaeR bound to the *salX* promoter and activated its transcription, and deletion-mapping electrophoretic mobility shift assays localized a putative BaeR-responsive sequence to 5′-TTCTTTTT-3′ within the −10/−35 spacer region. Similar to the *ΔbaeS/baeR* mutant, the *salX* mutant exhibited reduced resistance to human defensin 5 and impaired intestinal colonization. L-arginine exposure was associated with dose-dependent activation of the BaeS/BaeR pathway and BaeS/BaeR-dependent induction of *salX*, although a direct physical interaction between L-arginine and BaeS was not established.

**Discussion:**

These findings identify an L-arginine-associated BaeS/BaeR–SalX regulatory pathway that promotes antimicrobial peptide resistance and intestinal colonization by *V. cholerae*, providing new insight into host cue-associated regulation of bacterial colonization fitness.

## Introduction

*Vibrio cholerae* (*V. cholerae*) is the causative agent of cholera, causing approximately 3 to 5 million cholera infections and tens of thousands of deaths annually worldwide (Hu et al., 2016; Weill et al., 2019). The O1 El Tor biotype has driven the ongoing seventh cholera pandemic since its emergence in 1961. As a natural inhabitant of aquatic environments, *V. cholerae* alternates between environmental reservoirs and the human intestinal tract (Reidl and Klose, 2002; Temre et al., 2026). Following ingestion through contaminated food or water, the bacterium colonizes the distal small intestine and expresses major virulence factors, including cholera toxin (CT) and toxin-coregulated pilus (TCP) (Herrington et al., 1988; Teschler et al., 2015; Zingl et al., 2026). Successful colonization requires rapid sensing and adaptation to host-derived signals such as bile salts, osmotic stress, oxygen gradients, and pH fluctuations (Typas et al., 2011; Hsiao and Zhu, 2020; Bueno et al., 2022; Bosire et al., 2025; Ng'ombe et al., 2025; Alcaide-Jiménez et al., 2026). In common with many enteric pathogens, *V. cholerae* relies heavily on two-component systems (TCSs) to coordinate adaptive responses to these environmental cues (Nguyen et al., 2025).

Two-component systems (TCSs) are conserved bacterial signaling modules composed of a membrane-anchored histidine kinase (HK) and a cytoplasmic response regulator (RR), in which the HK senses environmental signals and initiates signaling via autophosphorylation (Schaefers, 2020; Sankhe et al., 2023). *V. cholerae* encodes multiple TCSs that mediate responses to diverse environmental and host-specific cues, including pH, oxygen, quorum sensing, and host-derived molecules (Wang et al., 2025). Several well-characterized systems, including PhoR/PhoB, EnvZ/OmpR, CitA/CitB, NtrB/NtrC, TorS/TorR, and ArcB/ArcA, contribute to biofilm formation, virulence regulation, and intestinal colonization (Mey et al., 2024). BaeS/BaeR is a conserved two-component system that has been implicated in envelope stress responses and antimicrobial resistance in several Gram-negative pathogens, including *Escherichia coli* and *Salmonella enterica*. In *Escherichia coli*, BaeS/BaeR functions as an envelope stress-responsive regulatory system that can be activated by indole, heavy metals, and envelope-damaging compounds, leading to BaeR-dependent induction of multidrug efflux systems such as *mdtABC* and *acrD*. Similar stress-adaptive functions have also been reported in *Salmonella enterica*. However, the role of BaeS/BaeR in sensing host-derived signals and regulating intestinal colonization remains poorly understood, particularly in *V. cholerae* (Nishino et al., 2005; Appia-Ayme et al., 2011).

To survive host-associated stresses such as antimicrobial peptides and antibiotics, bacteria employ multiple defense strategies, including active efflux systems and plasmid-mediated resistance mechanisms (Breland et al., 2017). ATP-binding cassette (ABC) transporters, as important efflux systems, play a key role in the export of bacteriocins and the active efflux of antibiotics (Rihacek et al., 2023). In several bacterial pathogens, TCSs functionally cooperate with ABC transporters to sense cell envelope stress and activate antimicrobial defense pathways (Duman et al., 2025; Hu et al., 2025). ABC transporters constitute a highly conserved protein superfamily that can mediate ATP-dependent translocation of diverse substrates—including peptides, amino acids, sugars, lipids, and antimicrobial compounds—and play important roles in nutrient acquisition, maintenance of cellular homeostasis, and resistance to antimicrobial peptides and antibiotics (Tomii and Kanehisa, 1998; Yakushi et al., 2000; Lee et al., 2007; Rempel et al., 2019; Luisi, 2025; Yu et al., 2026). Increasing evidence indicates that ABC transporters frequently form sensory-effector regulatory networks with two-component systems (TCSs), enabling bacteria to rapidly adapt to host-derived antimicrobial stress (Wilson, 2016). Representative examples include the BceRS–BceAB system in *Bacillus subtilis (*Chen and Kuipers, 2022) and the GraRS–VraFG system in *Staphylococcus aureus (*Rajagopal et al., 2016). However, whether similar TCS–ABC transporter regulatory circuits contribute to host adaptation and antimicrobial resistance in *V. cholerae* remains poorly understood.

BaeS (VCA0211) and BaeR (VCA0210), encoded by adjacent genes, form a putative TCS with homology to canonical bacterial histidine kinases and response regulators. Although homologous systems in other bacteria are associated with stress adaptation, the role of the BaeS/BaeR system in *V. cholerae* colonization, virulence regulation, and host cue-associated signaling remains unclear. In this study, we show that extracellular L-arginine functions as an upstream cue associated with BaeS/BaeR pathway activation and BaeR-dependent induction of the ABC transporter gene *salX*. This regulatory pathway enhances antimicrobial peptide resistance and intestinal colonization, revealing a mechanism by which *V. cholerae* couples environmental metabolic cues to innate immune evasion.

## Materials and methods

### Bacterial strains, plasmids, and culture conditions

All bacterial strains and plasmids used in this study are detailed in **Supplementary Tables 2**, **3**. The *V. cholerae* O1 El Tor strain EL2382 was obtained from the Shanghai Center for Disease Control and Prevention. *Escherichia coli* S17-1/λpir was utilized as the donor strain for conjugation-based mutagenesis, while *E. coli* BL21(DE3) served as the host for heterologous protein expression. Cultures were grown in Luria-Bertani (LB) broth or M9 minimal medium. Aerobic cultivation was performed at 37 °C with shaking at 180 rpm. Anaerobic cultures were incubated at 37 °C in an anaerobic chamber (YQX-II, Shanghai, China) under N_2_ atmosphere. Antibiotics were supplemented at the following concentrations when necessary: polymyxin B (40 μg/mL), ampicillin (50 μg/mL), and chloramphenicol (25 μg/mL).

### Mutant construction and complementation

The primer sequences employed in this investigation are detailed in **Supplementary Table 3**. Mutant strains, specifically Δ*baeS/baeR*, Δ*salX*, and Δ*baeR*/*salX*, were constructed via the suicide vector pRE112, employing a previously established protocol (Liu et al., 2021). In the context of ChIP–qPCR analyses, the *baeR* locus—encompassing its promoter—was inserted into pBAD33 to generate a C-terminally 3×Flag-tagged BaeR fusion protein. For recombinant BaeR production, the *baeR* gene was subcloned into the pET28a expression vector and heterologously expressed in *E. coli* BL21(DE3) cells.

### RNA isolation and qRT-PCR

Total RNA was extracted from collected biological specimens using the TransGen Biotech RNA Extraction Kit (Beijing, China). The integrity, concentration, and purity of RNA extracts were quantified spectrophotometrically via a NanoDrop 2000 system (Thermo Fisher Scientific), with absorbance ratios at 260/280 nm assessed to validate sample suitability for downstream analyses. First-strand complementary DNA (cDNA) synthesis was subsequently performed using the PrimeScript Reverse Transcription Reagent Kit (Thermo Fisher Scientific), following the manufacturer’s protocol with purified total RNA as template. Quantitative real-time PCR (qRT-PCR) assays were conducted by combining cDNA templates with SYBR Green Master Mix (Applied Biosystems) and gene-specific primer pairs. Amplification reactions were executed on an ABI 7500 Real-Time PCR System (Applied Biosystems), with cycling conditions optimized according to primer annealing temperatures. Relative transcript abundances of target genes were normalized against the endogenous reference gene 16S rRNA, and fold-changes in gene expression were calculated using the 2^−ΔΔCt^ method, where ΔCt represents the difference between Ct values of target and reference genes, and ΔΔCt denotes normalization against calibrator samples.

### RNA-seq

Overnight cultures of the WT and ΔbaeS/baeR strains were diluted 1:100 into fresh LB medium and grown to the mid-logarithmic phase (OD600 ≈ 0.6). Cells were harvested by centrifugation, and total RNA was extracted using TRIzol reagent according to the manufacturer’s instructions. Three independent biological replicates were prepared for each strain. RNA quality was assessed prior to library construction. Library preparation and paired-end sequencing were performed by Genewiz Inc. (Suzhou, China) using the Illumina platform. Raw sequencing reads were subjected to quality control to remove adapter sequences and low-quality reads, generating clean reads for subsequent analyses. Clean reads were aligned to the V. cholerae reference genome using Bowtie 2, and gene expression levels were quantified as fragments per kilobase of transcript per million mapped reads (FPKM). Differentially expressed genes (DEGs) were identified using the edgeR package following TMM normalization, with significance thresholds of |log_2_(fold change)|> 1 and a false discovery rate (FDR)-adjusted P value < 0.05, calculated using the Benjamini–Hochberg method.

### Growth assay

Bacterial proliferation in strains harboring *baeS/baeR* and *salX* deletions was evaluated by growth curve analysis. Following a 1:1000 dilution of overnight cultures into LB medium, the suspension was dispensed into 96-well plates (200 µL/well) and incubated at 37 °C with shaking (180 rpm). Optical density at 600 nm (OD600) was measured every 20 min continuously from 0 h to 18 h without repeated sample harvesting.

### Caco-2 cell adhesion assay

Caco-2 cells were acquired from the Shanghai Institute of Biochemistry and Cell Biology (Shanghai, China). The Caco-2 cell adhesion assay was implemented following a standardized method (Kozak et al., 2015). Specifically, Caco-2 monolayers were generated in 6-well plates and incubated in DMEM(HyClone Catalog No. SH30022.01) supplemented with 10% FBS for ≥16 hours. Before infection, the cells underwent three washes with pre-warmed PBS, and the medium was exchanged for serum- and antibiotic-free DMEM. If required, HD-5 was added to yield a final concentration of 50 μg/mL.

Bacterial cultures were subcultured in LB broth and incubated until reaching mid-logarithmic phase (OD_600_ value ≈ 0.6). Following three washes with PBS, bacteria were inoculated onto confluent Caco-2 monolayers at a multiplicity of infection (MOI) of 100 and co-incubated for 4 hours in Dulbecco’s Modified Eagle Medium (DMEM). Unbound bacteria were eliminated via extensive PBS rinsing, after which epithelial cells were lysed by incubation with 1 mL of 0.1% (v/v) Triton X-100 for 10 min at room temperature. The resultant cell lysates were serially diluted in PBS, plated onto LB agar supplemented with polymyxin B (50 μg/mL), and incubated overnight at 37 °C. Bacterial adhesion efficiency was subsequently determined by quantifying colony-forming units (CFU) per well.

### Electrophoretic mobility shift assay

Recombinant BaeR protein, engineered to carry a C-terminal 6×His tag, was expressed in *E. coli* BL21(DE3) and subsequently purified via affinity chromatography. A DNA fragment corresponding to the *salX* promoter was amplified by PCR and purified using the SPARKeasy Gel DNA Extraction Kit (catalog number AE0101; Sparkjade). For electrophoretic mobility shift assay (EMSA), 40 ng of purified *salX* promoter DNA was incubated with increasing concentrations of BaeR protein (0–0.8 μM) in a binding buffer composed of 10 mM Tris-HCl (pH 7.5), 5 mM MgCl2, 10 mM KCl, 0.3 mM dithiothreitol, and 15% (v/v) glycerol, with a total reaction volume of 20 μL. AcP (Acetyl phosphate) was supplemented in the above binding system at a final concentration of 2 mM to simulate the intracellular physiological environment. Following incubation, protein–DNA complexes were resolved on 8% pre-cooled native polyacrylamide gels at 4 °C under non-denaturing conditions and electrophoresed at 90 V. DNA–protein complexes were visualized by staining with 0.1% (w/v) GelRed and detected under UV illumination.

### ChIP-qPCR

Chromatin immunoprecipitation assays were performed essentially following established protocols with minor modifications (Li et al., 2020). Briefly, bacterial cultures were grown at 37 °C to mid-log phase (OD_600_ ≈ 0.3), after which protein expression was induced with 0.1 mM IPTG for 1 h. Protein–DNA interactions were stabilized by treatment with formaldehyde for 25 min, and crosslinking was quenched by the addition of glycine for 5 min at room temperature. Cells were harvested by centrifugation and resuspended in lysis buffer containing Tris-HCl (50 mM, pH 7.5), NaCl (100 mM), EDTA (1 mM), protease inhibitors, and lysozyme (20 mg/mL), followed by incubation at 37 °C to facilitate cell disruption. Chromatin was subsequently sheared by sonication to generate DNA fragments averaging approximately 500 bp in length. Clarified lysates were incubated with anti-3×Flag antibody (Sigma-Aldrich, F1804) in the presence of Protein A magnetic beads (Thermo Fisher Scientific, 10002D) to immunoprecipitate Flag-tagged protein–DNA complexes. Following extensive washing, crosslinks were reversed, and samples were treated with proteinase K and RNase A prior to DNA purification using a commercial PCR cleanup kit (SKJ-PP01, Sikejie Biotech). Mock immunoprecipitation samples processed in parallel without antibody served as negative controls. Enriched DNA fragments were quantified by qPCR using primers targeting the salX promoter, with the rpoS region used as a negative-control locus.

### Western blotting

Protein lysates were prepared from bacterial cultures grown in LB medium, Caco-2 cells following infection, and intestinal tissue samples. Samples underwent cell lysis followed by centrifugation clarification at 12,000 g for 15 min to isolate soluble protein fractions. These fractions were combined with 5× SDS-PAGE loading buffer (Thermo Fisher Scientific, Cat# NP0007) and denatured by incubation at 95 °C. Which 20 μg total protein was loaded per lane were separated via electrophoresis on 10% SDS-polyacrylamide gels and subsequently transferred to 0.22 μm polyvinylidene fluoride (PVDF) membranes using standard wet transfer protocols. Membranes were blocked with 5% BSA (Solvay, Catalog No. A8020.) in Tris-buffered saline containing Tween-20 (TBST) for 1 hour at room temperature to minimize non-specific binding. Primary antibody incubations were performed overnight at 4 °C with gentle agitation: mouse monoclonal anti-FLAG M2 antibody (Sigma-Aldrich, Cat# F1804; 1:1000 dilution) and rabbit polyclonal anti-RNA polymerase β subunit, RpoB, antibody (Abcam, Cat# ab191598; 1:1000 dilution). Following three washes with TBST (5 minutes each), membranes were incubated with horseradish peroxidase (HRP)-conjugated goat anti-rabbit IgG secondary antibody (Abcam, Cat# ab205718; 1:5000 dilution) for 1 hour at room temperature. Immunoreactive bands were detected using enhanced chemiluminescence (ECL) substrate and imaged with a ChemiDoc MP imaging system (Bio-Rad). Band intensities were quantified using ImageJ software (NIH), with values normalized to the RNA polymerase β loading control to account for inter-sample variability.

### Histological analysis

For histopathological evaluation, intestinal tissues were isolated from infected mice. Post-collection, samples underwent fixation in 4% paraformaldehyde (pH 7.4) for 48 hours, followed by paraffin embedding and sectioning (5 μm). Sections were deparaffinized in xylene and rehydrated through a graded ethanol series prior to H&E staining for morphological characterization.

### Animal experiments

To evaluate intestinal colonization capacity, five-day-old CD-1 neonatal mice were maintained under specific pathogen–free conditions in a temperature-controlled incubator (30 °C) with a 12 h light/dark cycle and relative humidity of 50 ± 5%. Competitive infection experiments were conducted with minor procedural adjustments to established protocols. Overnight cultures of *V. cholerae* strains carrying either the *lacZ* reporter (wild-type or mutant derivatives) or the corresponding Δ*lacZ* reference strain were grown in aerated Luria–Bertani medium at 37 °C. Briefly, *V. cholerae lacZ−* (Δ*lacZ*) strains and *lacZ+* (wild-type and mutant) strains were cultured overnight at 37 °C with aeration in Luria-Bertani (LB) broth, (approximately 1 × 10^5^ CFU each) were combined and orally administered to anesthetized pups (n = 6 per group). At the designated endpoint, the entire small intestine was excised, weighed, and mechanically homogenized in sterile buffer. Serial dilutions were plated on LB agar supplemented with X-gal to distinguish competing populations. Bacterial recovery was quantified based on colony counts, and the competitive index (CI) was calculated as the ratio of *lacZ+* to *lacZ−* cells recovered from the small intestine, normalized to the corresponding ratio in the inoculum.

### Statistical analyses

All statistical analyses were performed using GraphPad Prism (v9.0.1; GraphPad Software, San Diego, CA, USA). The number of independent replicates and the statistical tests used are indicated in the corresponding figure legends. Data were first tested for normality using the Shapiro–Wilk test. Normally distributed data were analyzed using an unpaired Student’s t-test, whereas non-normally distributed data were analyzed using the Mann–Whitney U test. Comparisons among three or more groups were performed using one-way or two-way analysis of variance (ANOVA), followed by Dunnett’s multiple-comparisons test where appropriate. Survival curves were compared using the log-rank (Mantel–Cox) test. A P value less than 0.05 was considered statistically significant.

## Results

### BaeS/BaeR TCS promotes intestinal colonization of *V. cholerae*

**Figure 1:**
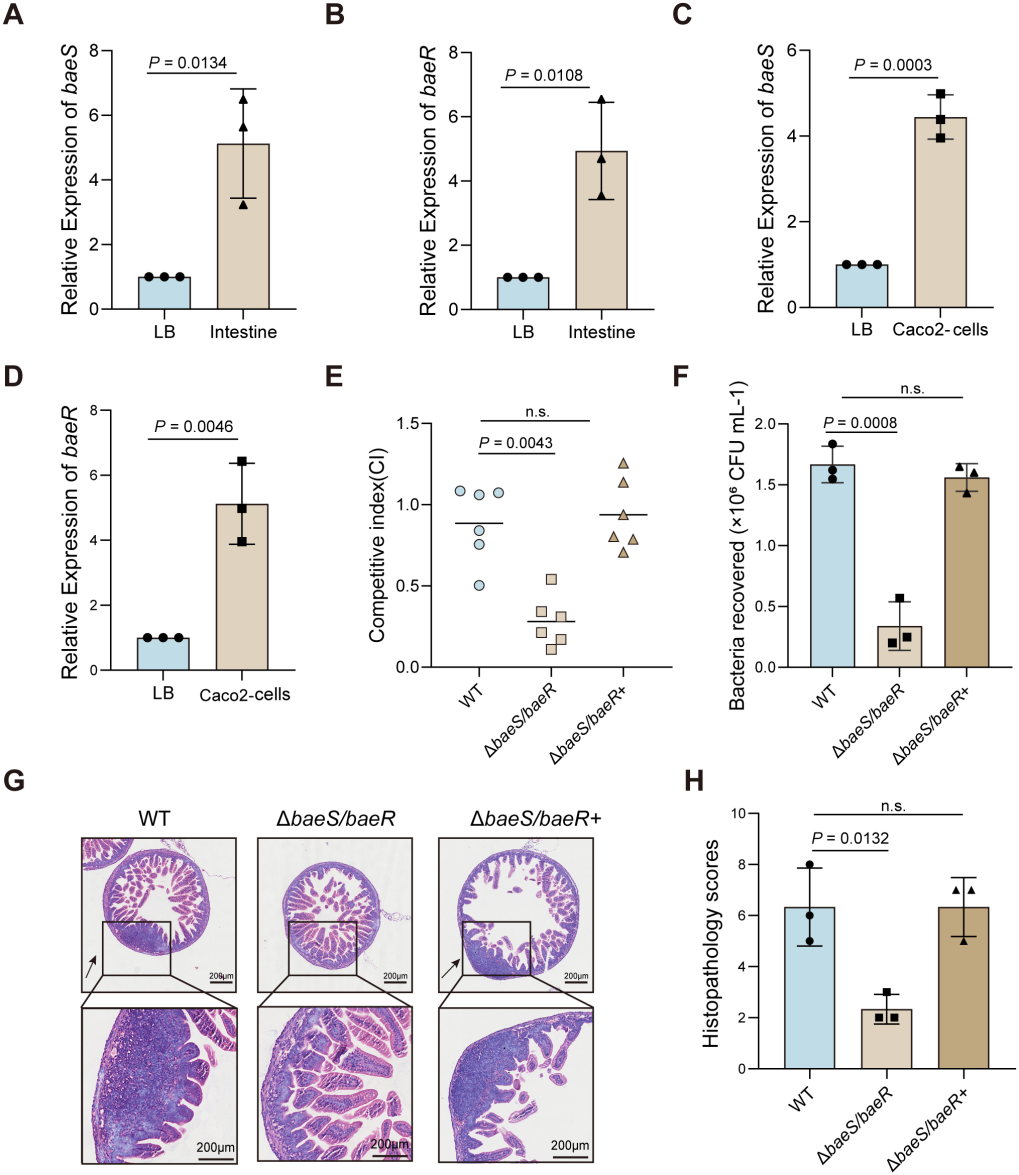
BaeS/BaeR TCS promotes intestinal colonization of *V. cholerae*. **(A, B)** Relative transcript levels of *baeS* **(A)** and *baeR* **(B)** were quantified by qRT-PCR in the small intestines of neonatal mice infected with *V. cholerae* and in bacteria grown in LB medium (*n* = 3). **(C, D)** mRNA expression of *baeS* **(C)** and *baeR* **(D)** in WT *V. cholerae* 4 hours post-infection of Caco-2 cells, compared to LB controls (*n* = 3). **(E)** Competitive colonization of neonatal mice by WT, Δ*baeS*/*baeR*, and Δ*baeS*/*baeR* +. The competitive index (CI) was calculated as the output ratio of mutant to WT *lacZ−* bacteria normalized to the corresponding input ratio. Each data point represents one mice (*n* = 6). **(F)** Adhesion capacity of WT, Δ*baeS*/*baeR* and Δ*baeS*/*baeR*+ strains to Caco-2 cells at 4 hours post-infection (*n* = 3). **(G)** Histopathological representation and **(H)** corresponding histological scores of WT, *ΔbaeS/baeR*, and *ΔbaeS/baeR*+ strains in the small intestines of neonatal mice 24 h post-infection (*n* = 3). The lower panels are higher-magnification images of the boxed regions, showing inflammatory cell infiltration (arrows). Results are presented as mean ± SD from three independent experiments. Differences between groups were evaluated by unpaired Student’s *t* test or Mann–Whitney U test. *P* values are indicated; n.s., not significant.

Previous studies have demonstrated an uneven distribution of the BaeS/BaeR two-component system between pandemic and non-pandemic *V. cholerae* strains, with its presence predominantly limited to pandemic variants (Wang et al., 2025). To investigate the potential role of BaeS/BaeR in intestinal colonization, we quantified the expression of BaeS/BaeR via quantitative reverse transcription PCR (qRT-PCR) in bacteria isolated from the small intestines of infected neonatal mice and compared them to bacteria cultured in Luria-Bertani (LB) broth (**Figures 1A, B**). To further evaluate BaeS/BaeR expression under host-associated conditions, we compared the transcription levels of *baeS* and *baeR* in wild-type (WT) *V. cholerae* grown in LB medium and after adhesion to Caco-2 cells. As shown in **Figures 1C, D**, both genes were significantly upregulated following 4 h of adhesion, indicating that the BaeS/BaeR system is activated during intestinal epithelial cell adhesion.

To elucidate the functional contribution of the BaeS/BaeR TCS to *V. cholerae* intestinal colonization, we conducted experiments using a neonatal mouse model. A *baeS/baeR* double-mutant strain (Δ*baeS/baeR*) and its genetically complemented derivative (Δ*baeS/baeR*+) were engineered in the genetic background of the seventh-pandemic *V. cholerae* strain EL2382. Competitive colonization assays showed that the intestinal colonization capacity of the Δ*baeS/baeR* mutant was significantly reduced compared with that of the WT strain, whereas no significant difference was observed between the complemented strain (Δ*baeS/baeR*+) and the WT strain. These results support our hypothesis that the BaeS/BaeR two-component system promotes the intestinal colonization of *V. cholerae* in the small intestine (**Figure 1E**). To rule out growth rate variations as a confounding variable, we performed anaerobic growth curve analyses for WT, Δ*baeS/baeR*, and Δ*baeS/baeR*+ strains. No statistically significant differences in growth kinetics were observed among these strains *in vitro* (**Supplementary Figure 1**), indicating that the BaeS/BaeR TCS modulates intestinal colonization independently of bacterial proliferation. Adhesion assays using Caco-2 cells demonstrated that both *baeS* (sensor kinase) and *baeR* (response regulator) single mutants exhibited markedly reduced adherence to host cells compared to WT (**Figure 1F**). Histopathological evaluation of neonatal mice small intestines via hematoxylin and eosin (H&E) staining revealed that infection with Δ*baeS*/*baeR* induced significantly lower pathological scores relative to WT infection (**Figures 1G, H**). Collectively, these findings establish the BaeS/BaeR TCS as a critical determinant of intestinal colonization fitness and pathogenic potential in *V. cholerae*.

### BaeS/BaeR TCS upregulates *salX* expression in *V. cholerae*

**Figure 2:**
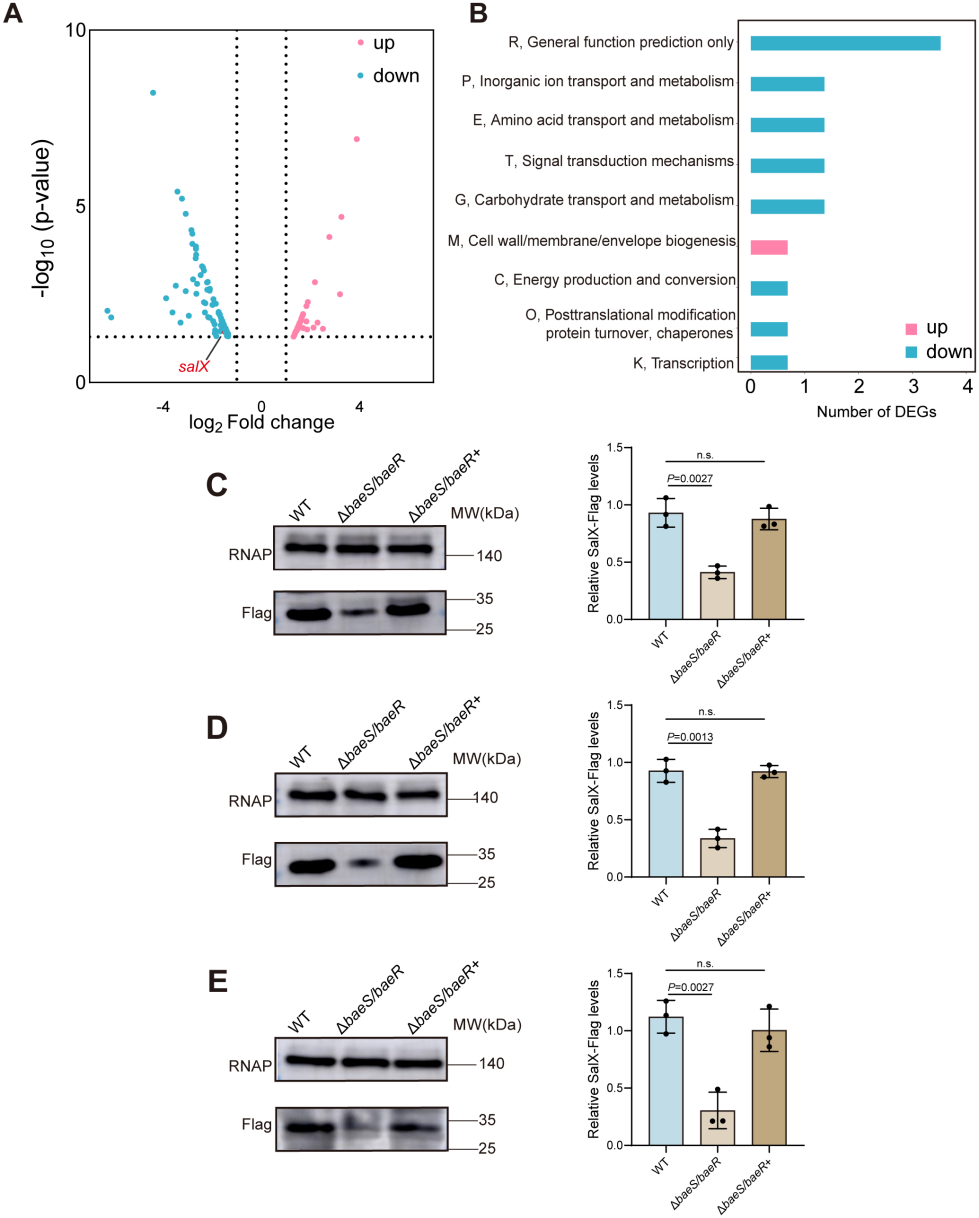
BaeS/BaeR TCS upregulates *salX* expression in *V. cholerae*. **(A)** Volcano plot showing transcriptional changes in the Δ*baeS*/*baeR* relative to the WT strain grown in LB. The X-axis indicates log_2_-fold change, and the Y-axis represents −log_10_ (*P*-value). Differentially expressed genes were identified using edgeR following TMM normalization, with P values adjusted using the Benjamini–Hochberg false discovery rate method. **(B)** Functional classification of differentially regulated genes based on COG categories in the Δ*baeS*/*baeR*. Bars indicate the number of genes exhibiting increased (red) or decreased (light blue) expression. Enrichment within each COG category was evaluated using a hypergeometric test. **(C)** Western blot analysis of SalX protein abundance in WT, Δ*baeS*/*baeR* and Δ*baeS*/*baeR*+ strains cultured in LB medium. **(D)** SalX protein levels in WT, Δ*baeS*/*baeR* and Δ*baeS*/*baeR*+ strains after 4 h interaction with Caco-2 cells, determined by Western blotting. **(E)** Detection of SalX expression in the small intestines of neonatal mice infected with WT, Δ*baeS*/*baeR* and Δ*baeS*/*baeR*+ strains following oral inoculation. RNAP served as the loading control (*n* = 3). Quantitative data are presented as mean with SD from three independent experiments. Statistical comparisons for **(C–E)** were performed using an unpaired Student’s *t*-test. Exact *P* values are indicated, and n.s. denotes a non-significant difference.

To investigate the mechanism by which the BaeS/BaeR TCS regulates the pathogenicity of *V. cholerae*, we performed RNA-seq analysis of the WT and *ΔbaeS/baeR* strains cultured in LB medium. Transcriptomic analysis identified a total of 126 differentially expressed genes, including 42 upregulated and 84 downregulated genes (**Supplementary Table 1**). Notably, the transcriptional level of the *salX* gene was downregulated in the Δ*baeS/baeR* strain (**Figure 2A**).

SalX is a component of an ABC-type antimicrobial peptide transporter system that plays a role in bacterial colonization. The differentially expressed genes were categorized based on the NCBI Clusters of Orthologous Groups (COG) functional classification system. Analysis revealed that upregulated genes were predominantly enriched in functional categories associated with signal transduction mechanisms and cell envelope biogenesis. Conversely, downregulated genes exhibited significant enrichment in functions related to inorganic ion transport, amino acid and carbohydrate metabolism, energy production, signal transduction, and protein turnover (**Figure 2B**). To determine the expression profile of *salX* under host-mimicking conditions, WT and Δ*baeS*/*baeR* strains were cultured in LB broth, followed by Caco-2 cell adhesion assays and neonatal mice gavage experiments. qRT-PCR analysis revealed that the two-component system BaeS/BaeR functions as a positive regulator of *salX* expression (**Supplementary Figure 2**). Western blot analyses further demonstrated that under LB culture conditions (**Figure 2C**), SalX protein levels were markedly reduced in the Δ*baeS*/*baeR* strain compared with WT. Following incubation with Caco-2 cells (**Figure 2D**), SalX expression was likewise substantially decreased in the Δ*baeS*/*baeR* strain. Moreover, in the neonatal mice infection model (**Figure 2E**), SalX expression in the small intestine was also significantly lower in the Δ*baeS*/*baeR* than in WT strain. In contrast, SalX expression in the complemented strain was restored to levels comparable to those observed in WT strain. Collectively, these findings indicate that BaeS/BaeR positively regulates SalX expression under both *in vitro* and *in vivo* conditions.

### BaeS/BaeR TCS promotes *V. cholerae* intestinal colonization and HD-5 resistance by regulating *salX*

**Figure 3:**
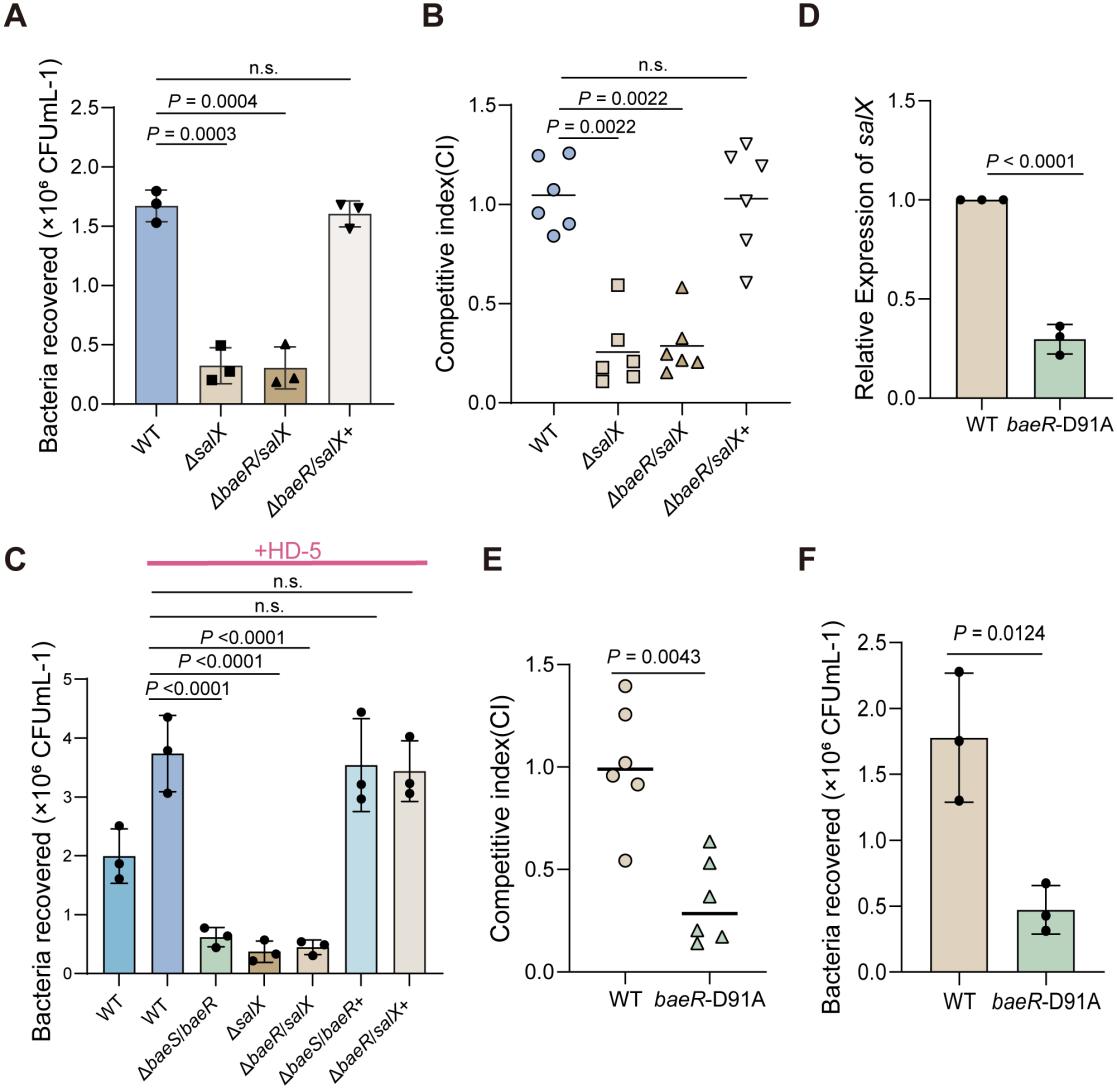
BaeS/BaeR TCS promotes *V. cholerae* intestinal colonization and HD-5 resistance by regulating *salX*. **(A)** Adhesion of WT, Δ*salX*, Δ*baeR/salX* and Δ*baeR/salX*+ strains to Caco-2 cells was quantified after 4 hours of incubation and bacterial counting (*n* = 3). **(B)** Competitive infection assay in mice measured the bacterial load of WT, Δ*salX*, Δ*baeR/salX*, and Δ*baeR/salX+* strains in the small intestine 24 hours post-inoculation (*n* = 6). **(C)** Adhesion of *V. cholerae* strains to Caco-2 cells was measured in the presence of 50 μg/mL HD-5 antimicrobial peptide after 4 hours of incubation (*n* = 3). **(D)** Analysis of *salX* transcription in the *baeR*-D91A phosphorylation-site mutant (*n* = 3). **(E)** Competitive infection assay in mice measured the bacterial load of WT and *baeR*-D91A strains in the small intestine 24 hours post-inoculation (*n* = 6). **(F)** Adhesion assay evaluating the attachment of the *baeR*-D91A mutant and WT strain to Caco-2 cells (*n* = 3). Data are shown as mean ± SD from three independent experiments. Statistical significance was assessed using unpaired Student’s *t*-test or Mann–Whitney U test. Exact *P* values are indicated; n.s., not significant.

We next investigated whether the impact of BaeS/BaeR on *V. cholerae* colonization-associated phenotypes is related to its regulation of *salX* expression. Adhesion assays revealed that *salX* contributes to BaeS/BaeR-mediated host-cell adhesion. The Δ*salX* and Δ*baeR/salX* strains exhibited significantly reduced adhesion to Caco-2 cells compared to the WT strain, whereas the complemented strain (Δ*baeR*/*salX*+) restored adhesion to levels comparable to the WT strain (**Figure 3A**). Competitive infection assays conducted in the small intestine of mice showed that the competitive indices of the Δ*salX* and Δ*baeR*/*salX* strains were significantly lower than those of the WT and Δ*baeR*/*salX*+ strains (**Figure 3B**). To account for potential growth-related effects, the anaerobic growth kinetics of the bacterial strains were assessed *in vitro*. No significant differences in growth rates were observed among the WT, Δ*salX*, Δ*baeR/salX*, and Δ*baeR/salX*+ strains (**Supplementary Figure 3**). Moreover, Kaplan–Meier survival analysis showed that mice infected with the Δ*baeS*/*baeR*, Δ*salX*, or Δ*baeR*/*salX* mutant strains exhibited improved survival compared with WT-infected mice, whereas complementation restored the survival phenotype toward that of the WT-infected group (**Supplementary Figure 9**). Collectively, these data demonstrate that *salX* contributes to intestinal colonization by *V. cholerae* independently of its effect on bacterial growth.

In *Bacillus subtilis*, exposure to extracellular antimicrobial agents like bacitracin induces expression of the ABC transporter genes *bceAB*, which confers resistance against diverse antimicrobial peptides (AMPs), including fungal defensins such as plectasin, gardimycin, actagardine, and mersacidin (Rismondo and Schulz, 2021; George et al., 2022). Consistent with this function, deletion mutants lacking functional *bceAB* exhibit hypersensitivity to bacitracin, underscoring its essential role in mediating bacitracin resistance (Cho et al., 2021). Human defensin 5 (HD-5), a principal AMP secreted by intestinal paneth cells, is critical for mucosal innate immunity (Liu Y. et al., 2022). Cell adhesion assays demonstrated that incubation with 50 μg/mL HD-5 significantly attenuated the adhesion capacity of Δ*baeS*/*baeR*, Δ*salX*, and Δ*baeR*/*salX* strains compared to controls (**Figure 3C**). These results indicate that disruption or downregulation of *salX* compromises *V. cholerae’s* resistance to HD-5. To directly assess HD-5 resistance, we further performed HD-5 killing assays and MIC measurements. The Δ*baeS*/*baeR*, Δ*salX*, and Δ*baeR*/*salX* mutants showed reduced survival and lower MIC values against HD-5 compared with the WT strain, whereas complementation restored HD-5 resistance (**Supplementary Figure 4**).

Since BaeR acts as a response regulator and its transcriptional activity is dependent on phosphorylation, we next investigated whether *salX* expression is regulated in a phosphorylation-dependent manner. Sequence alignment and domain analysis revealed that the conserved phosphorylation site is located at Asp91 within the receiver domain. Elimination of the phosphorylation site in the *baeR*-D91A mutant resulted in a marked suppression of *salX* transcription LB medium (**Figure 3D**). *In vivo* competitive infection assays demonstrated that, compared with the WT strain, the phosphorylation-defective mutant exhibited significantly impaired intestinal colonization (**Figure 3E**). Furthermore, adhesion assays showed that the *baeR*-D91A mutant had markedly reduced adherence to Caco-2 cells relative to the WT strain (**Figure 3F**). These observations are consistent with the phenotype of the Δ*baeS*/*baeR* strain, suggesting that Asp91 is required for BaeR-dependent regulation of *salX*. To further evaluate whether the D91A substitution affects SalX expression at the protein level, we performed Western blot analysis of SalX abundance in WT and baeR-D91A strains grown in LB medium. Consistent with the reduced *salX* transcription observed in the baeR-D91A mutant, SalX protein abundance was markedly decreased in the *baeR*-D91A background compared with the WT strain (**Supplementary Figure 5**). These results support the conclusion that Asp91 is important for BaeR-mediated activation of *salX* and for *V. cholerae* adhesion and small-intestinal colonization.

### BaeS/BaeR TCS directly binds to *salX*

**Figure 4:**
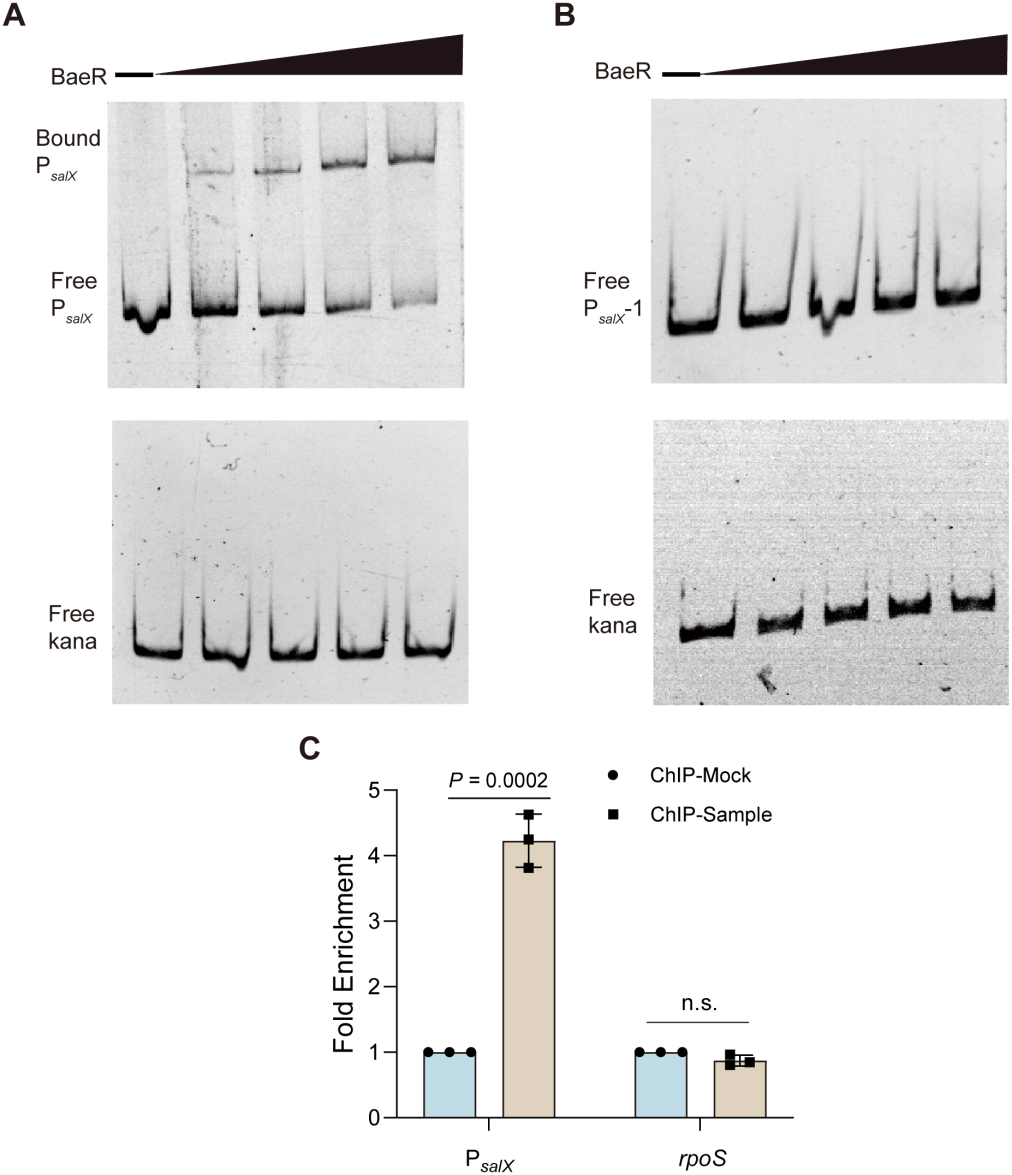
BaeS/BaeR TCS directly binds to *salX*. **(A)** Interaction between BaeR and the *salX* promoter was examined by EMSA using increasing amounts of purified BaeR protein. **(B)** EMSA using the P*_salX_* -1 promoter (lacking −10 and −35 spacer sequence) was performed to confirm binding specificity of BaeR to the *salX* promoter. **(C)** ChIP-qPCR analysis was conducted to assess the enrichment of the *salX* promoter in baeR-ChIP samples compared to mock ChIP samples (*n* = 3). Data represent the mean ± SD from three independent experiments. Statistical significance was evaluated using unpaired Student’s *t*-test; n.s., not significant.

To investigate whether the BaeS/BaeR two-component system regulates *V. cholerae* pathogenicity through direct binding to the *salX* promoter, we first performed bioinformatic analysis of the *salX* upstream regulatory region. This analysis identified a putative BaeR recognition motif. Electrophoretic mobility shift assays (EMSAs) were then conducted using purified His_6_-tagged BaeR protein. A dose-dependent mobility shift was observed for the *salX* promoter fragment as the concentration of BaeR increased from 0 to 0.8 µM (**Figure 4A**), indicating specific interaction between BaeR and the *salX* promoter.

In bacterial regulatory systems, transcription factors often bind to sequences within the spacer region separating the −10 and −35 promoter elements, where DNA structural modulation can facilitate RNA polymerase recruitment (Wu et al., 2022). To investigate whether this region is involved in BaeR-mediated regulation of the *salX* promoter, we constructed a truncated promoter fragment, P*_salX_*-1, lacking the −10/−35 spacer sequence (5’-CCGATAGTTTCTTTTT-3’), and performed EMSA analysis. The results showed that deletion of this spacer completely abolished BaeR–DNA complex formation (**Figure 4B**), indicating that this region is essential for BaeR interaction with the *salX* promoter. To further define the BaeR-binding sequence within this spacer, we divided the 16-bp spacer into two subregions and generated two additional deletion derivatives. In P*_salX_*-2, the first half of the spacer (5’-CCGATAGT-3’), was deleted, whereas in P*_salX_*-3, the second half (5’-TTCTTTTT-3’), was deleted. EMSA analysis showed that BaeR retained binding to the P*_salX_*-2 fragment, indicating that deletion of 5’-CCGATAGT-3’ did not disrupt BaeR–DNA complex formation. By contrast, deletion of (5’-TTCTTTTT-3’) in P*_salX_*-3 abolished the shifted BaeR–DNA complex, indicating that this region is required for BaeR binding to the *salX* promoter (**Supplementary Figure 8**). These results refine the candidate BaeR-responsive region and identify (5’-TTCTTTTT-3’) as the putative core sequence required for BaeR recognition. ChIP-qPCR analysis further showed a 4.23-fold enrichment of the *salX* promoter in BaeR-immunoprecipitated samples relative to mock controls (**Figure 4C**), supporting the *in vivo* association between BaeR and the *salX* promoter. Together, these findings demonstrate that BaeR directly engages the *salX* promoter to activate transcription, establishing *salX* as a downstream effector of the BaeS/BaeR regulatory pathway. A candidate BaeR-responsive region was localized to the spacer between the predicted −10 and −35 elements of the *salX* promoter. Deletion of this region abolished BaeR binding in EMSA assays, indicating that it is required for promoter recognition. Although site-directed mutagenesis identified this −10/−35 spacer sequence as a functional BaeR-responsive region required for BaeR binding and *salX* promoter activation, the precise protein–DNA contact nucleotides remain to be further defined by DNase I footprinting or structural analyses.

### L-arginine exposure is associated with BaeS/BaeR-dependent *salX* induction and enhanced intestinal colonization

**Figure 5:**
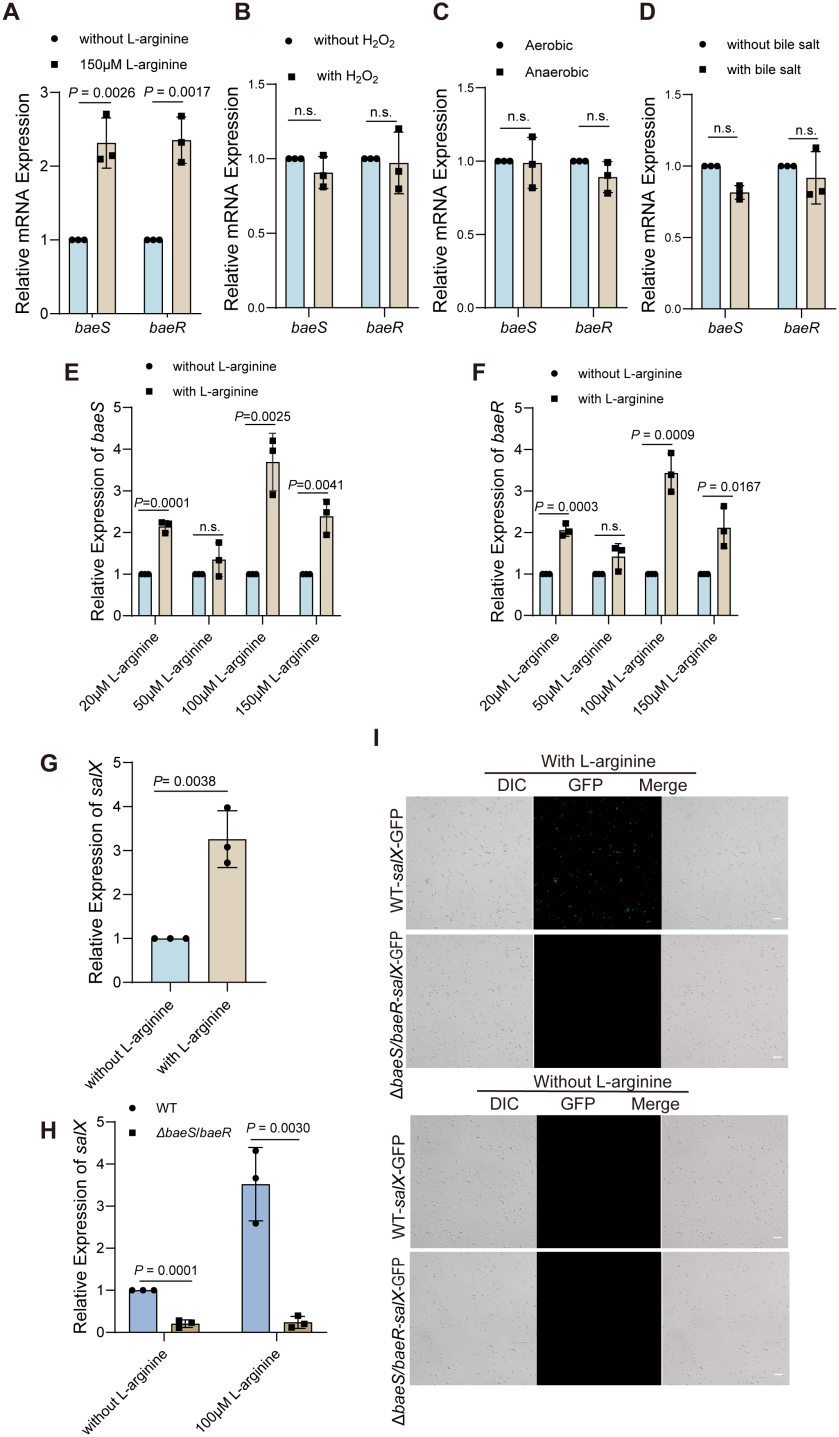
L-arginine exposure is associated with BaeS/BaeR-dependent salX induction and enhanced intestinal colonization. **(A-D)** Expression levels of *baeS* and *baeR* in WT *V. cholerae* under L-arginine **(A)**, oxidative stress (H_2_O_2_, 50 μM) **(B)**, aerobic or anaerobic condition **(C)** and bile salts (100 μM) **(D)**, measured by qRT-PCR (*n* = 3). **(E, F)** Dose-dependent expression of *baeS* **(E)** *and baeR* **(F)** in response to increasing concentrations of L-arginine (*n* = 3). **(G)** Expression level of *salX* transcripts in the WT strain cultured in M9 minimal medium with or without 100 μM L-arginine (*n* = 3). **(H)** Quantification of *salX* expression levels in WT and Δ*baeS*/*baeR* strains under L-arginine–supplemented and control condition (*n* = 3). **(I)** Confocal imaging of GFP fluorescence in WT and Δ*baeS*/*baeR* strains harboring a *salX* promoter–GFP transcriptional reporter, cultured in the presence or absence of 100 μM L-arginine. Data are shown as mean ± SD from three independent biological replicates. Statistical significance was evaluated using an unpaired Student’s *t*-test; n.s., not significant.

To investigate the potential role of the BaeS/BaeR TCS in enhancing *V. cholerae* intestinal colonization through modulation of host signal responses, we first considered the diverse intestinal environmental cues encountered by pathogenic bacteria during infection, including reactive oxygen species (ROS) (Yoon et al., 2016), oxygen fluctuations (Sun et al., 2022), bile salts (Zhang et al., 2024), and L-arginine (Klotz et al., 2026). This prompted us to assess whether the BaeS/BaeR TCS enables *V. cholerae* to respond and adapt to these colonization-relevant host-derived cues. To identify upstream regulatory stimuli, we quantified transcriptional changes of *baeS* and *baeR* in response to physiologically relevant intestinal signals: elevated ROS (50 μM), altered oxygen availability (aerobic vs. anaerobic conditions), bile salts (100 μM), and L-arginine (150 μM) (Ma et al., 2021; Li et al., 2025). qRT-PCR analysis revealed that variations in ROS levels, oxygen tension, and bile salt concentration had no significant impact on *baeS/baeR* mRNA abundance in the WT. In contrast, L-arginine exposure was associated with markedly increased transcript levels of both genes (**Figures 5A–D**).

Previous studies reported that the intestinal concentration of free L-arginine in adult mice is approximately 21.7 μM (Hou et al., 2020). However, suckling mice exhibit distinct intestinal amino acid metabolic characteristics compared with adult mice. To further evaluate the physiological relevance of our experimental conditions, we quantified free L-arginine levels in the intestinal lumen of both adult and suckling mice using HPLC analysis. As shown in **Supplementary Figure 6**, the luminal concentrations of free L-arginine in adult and suckling mice were 26.91 μM and 66.78 μM, respectively, indicating that suckling mice maintain substantially higher intestinal L-arginine levels.

Based on these observations, we further investigated the dose-dependent association between L-arginine exposure and *baeS/baeR* expression. Concentrations ranging from 20–150 μM effectively induced transcription of both genes, with significant induction already observed at 20 μM, indicating that the BaeS/BaeR pathway responds to physiologically relevant variation in L-arginine availability (**Figures 5E, F**). Among the tested concentrations, 100 μM produced the most stable and robust induction effect in bacteria cultured in M9 minimal medium and was therefore selected as the representative concentration for subsequent mechanistic analyses. Importantly, this concentration range overlaps with the physiological concentrations detected in infant mice and may also reflect transient local enrichment occurring within intestinal mucus layers or bacterial colonization niches during infection.

To determine whether the BaeS/BaeR TCS is required for L-arginine-associated regulation of *salX* expression, qRT-PCR was performed to quantify *salX* transcription in M9 minimal medium supplemented with 100 μM L-arginine. L-arginine exposure markedly induced *salX* transcription relative to the untreated control, consistent with the induction patterns observed for *baeS* and *baeR* (**Figure 5G**). To determine whether this induction depends on the BaeS/BaeR pathway, *salX* expression was further examined in the Δ*baeS*/*baeR* mutant following L-arginine treatment. In contrast to the WT strain, the Δ*baeS*/*baeR* mutant did not exhibit altered *salX* expression upon exposure to 100 μM L-arginine (**Figure 5H**). These results indicate that L-arginine-associated induction of *salX* requires the BaeS/BaeR TCS. To further examine whether L-arginine-associated *salX* induction depends on the conserved phosphorelay residues of BaeS/BaeR, we analyzed *salX* expression in the *baeS*-H410A and *baeR*-D91A mutant strains. L-arginine induced *salX* expression in a dose-dependent manner in the WT strain, whereas this response was abolished or markedly impaired in both mutant backgrounds (**Supplementary Figure 7**), indicating that intact BaeS/BaeR phosphorelay activity is required for L-arginine-mediated *salX* activation.

To confirm the qRT-PCR results, the *salX*-GFP transcriptional fusion plasmid (Leastro et al., 2025), containing the *salX* promoter fused to the GFP reporter gene, was generated and transformed into WT and Δ*baeS/baeR*. GFP fluorescence was assessed by confocal microscopy following growth in M9 medium with or without L-arginine. In the WT strain, GFP expression was observed only when cultured in M9 minimal medium with L-arginine. By contrast, fluorescence was absent in the Δ*baeS/baeR* strain irrespective of L-arginine supplementation (**Figure 5I**), indicating that BaeS/BaeR is required to transduce the L-arginine signal to the *salX* promoter. Together, these results identify L-arginine as an upstream cue that activates the BaeS/BaeR pathway, leading to the induction of *salX* and reinforcing host-associated fitness of *V. cholerae*.

## Discussion

Successful intestinal colonization by *V. cholerae* requires responsiveness to host-derived metabolic cues and rapid adaptation to immune-mediated stress. In this study, we identified a BaeS/BaeR-dependent regulatory cascade that links L-arginine exposure to transcriptional activation of the ABC transporter gene *salX*, thereby enhancing resistance to antimicrobial peptide (AMP)-mediated stress and promoting efficient colonization.

**Figure 6:**
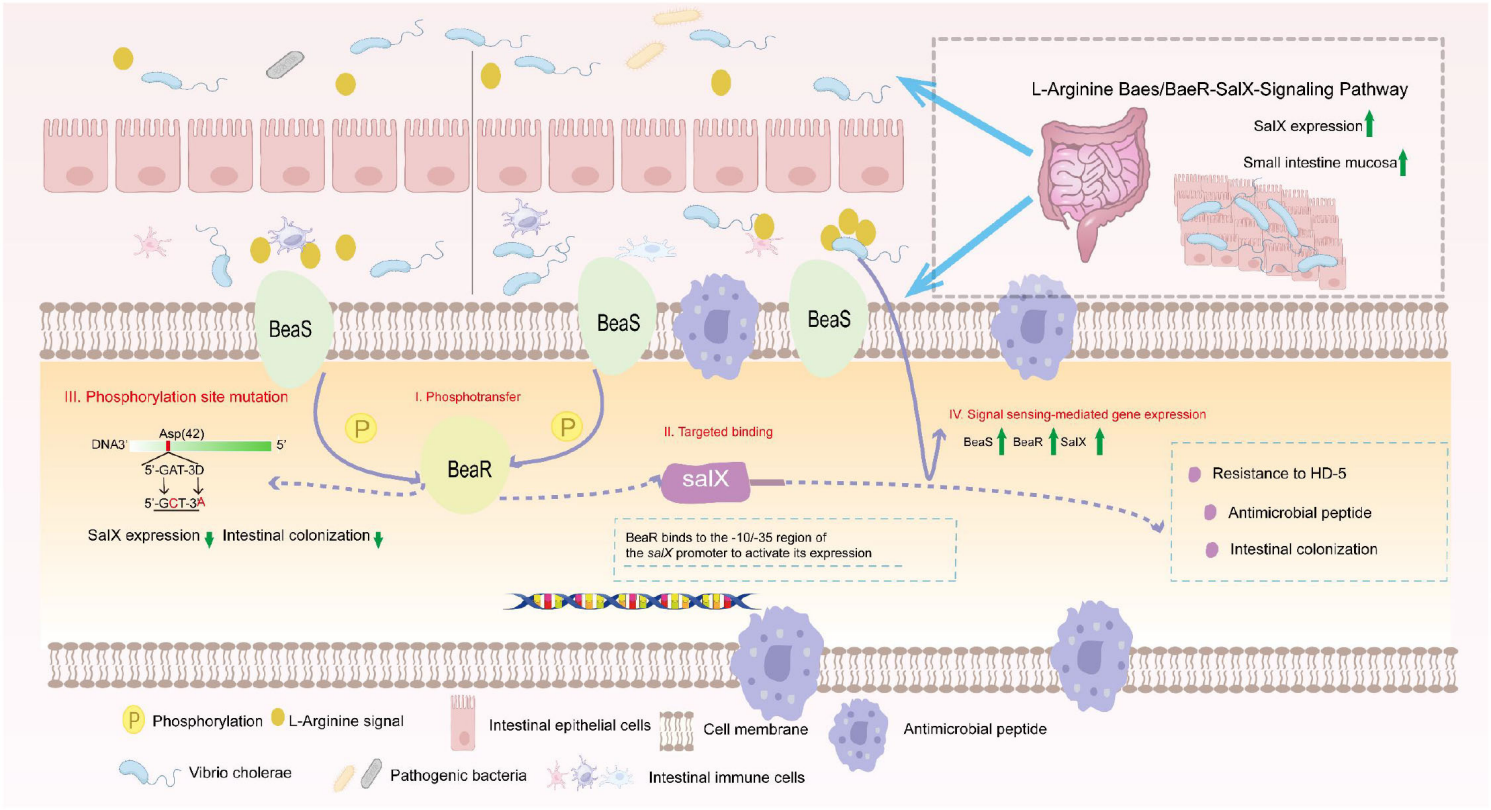
Schematic model of the L-arginine–BaeS/BaeR–SalX signaling pathway in *V. cholerae*. (By Figdraw). L-arginine exposure is associated with activation of the BaeS/BaeR pathway, inducing *salX* expression to enhance antimicrobial peptide resistance and intestinal colonization; the present study does not establish a direct L-arginine-BaeS interaction.

Mechanistically, BaeR directly binds to the *salX* promoter to activate its transcription, with phosphorylation at the conserved Asp91 residue in its receiver domain being essential for this activity. Phosphorylation at Asp91 is essential for BaeR-mediated activation of *salX* transcription. Deletion of the conserved phosphorylation site significantly reduces *salX* expression and impairs bacterial adhesion and intestinal colonization, demonstrating that phosphorylation-dependent signal transduction is critical for pathway functionality and intestinal colonization. Transcriptomic analyses further revealed that BaeS/BaeR regulates a broader set of genes involved in transport, metabolism, and stress adaptation, indicating that *salX* represents one important downstream target within a larger adaptive regulatory network. A schematic model summarizing the proposed BaeS/BaeR–SalX signaling pathway and its contribution to intestinal colonization is presented in **Figure 6**.

BaeS/BaeR is a highly conserved two-component system that has been extensively characterized in *Enterobacteriaceae* as a sensor of envelope-associated stress. In *Escherichia coli*, BaeS/BaeR is activated by diverse environmental insults, including indole, heavy metals, and envelope-damaging compounds, and subsequently induces the expression of multidrug efflux systems such as *mdtABC* and *acrD* to enhance bacterial stress tolerance and antimicrobial resistance (Nishino et al., 2005; Leblanc et al., 2011). Similar functions have also been reported in *Salmonella enterica*, where BaeS/BaeR contributes to envelope homeostasis and adaptation to hostile environments (Appia-Ayme et al., 2011). Despite the widespread distribution of BaeS/BaeR homologs among Gram-negative bacteria, previous studies have primarily linked this system to envelope stress responses and multidrug resistance. In contrast, little is known regarding its role in host-derived metabolic signal responsiveness and virulence regulation in *Vibrio* species. Our findings therefore expand the functional repertoire of BaeS/BaeR by demonstrating that this pathway responds to L-arginine-associated conditions and regulates SalX-dependent antimicrobial peptide resistance and intestinal colonization in *V. cholerae*, while leaving the direct molecular trigger of BaeS/BaeR activation unresolved. Notably, virulence-associated determinants of *Vibrio* can also persist in environmental and foodborne reservoirs. For example, multiple virulence-associated genes have been detected in *Vibrio* isolates recovered from leafy vegetables (Olawale Oladejo et al., 2025), suggesting that regulatory pathways controlling colonization-associated traits may also contribute to ecological adaptation outside the human host.

ABC transporters have emerged as well-established components of bacterial defense networks, particularly when integrated into TCS-regulated sensory–effector circuits that mediate antimicrobial peptide (AMP) resistance. Classic paradigms include the BceRS–BceAB system in *Bacillus subtilis* and the GraRS–VraFG system in *Staphylococcus aureus*, both of which confer enhanced envelope resilience under antimicrobial stress (Diagne et al., 2022; Orsini Delgado et al., 2024). In alignment with this mechanism, our findings reveal that the SalX-encoded ABC transporter constitutes a critical effector mediating resistance to the host-derived AMP HD-5 in *V. cholerae*. Genetic deletion of either *salX* or its cognate BaeS/BaeR TCS markedly impairs bacterial tolerance to HD-5 under the tested conditions, thereby positioning *salX* as an essential downstream effector of BaeS/BaeR-mediated antimicrobial defense during intestinal colonization.

Host and microbiota-derived metabolites have emerged as important environmental cues that shape bacterial pathogenicity. Recent studies demonstrated that enterohemorrhagic *Escherichia coli* senses microbiota-derived riboflavin (Liu B. et al., 2022) and nicotinamide (Yang et al., 2023) to activate virulence-associated pathways, while *V. cholerae* responds to human α-defensin 5 through the CarSR two-component system (Liu Y. et al., 2022) and to oxygen limitation through MlrA-dependent regulation (Wu et al., 2022). Unlike these previously described pathways, the signaling cascade identified here links responsiveness to a host-associated nutritional metabolite to activation of an antimicrobial peptide resistance determinant. L-arginine concentrations measured in the intestinal lumen of infant mice fall within the range capable of inducing *baeS/baeR* and *salX* expression, supporting its physiological relevance during infection. Moreover, significant induction of *baeS*, *baeR*, and *salX* was observed at concentrations as low as 20 μM, indicating that the BaeS/BaeR system is highly sensitive to fluctuations in intestinal L-arginine availability (Hou et al., 2020; Hernández et al., 2021; Kanda et al., 2025; Story et al., 2025). Although L-arginine is treated here as a host-derived metabolic cue, its local availability within the intestinal lumen is also influenced by the metabolic activity of the resident gut microbiota. Increasing evidence suggests that microbiota-derived metabolites and nutrient competition shape pathogen virulence programs and colonization resistance at the host–microbiota interface (Bayode et al., 2026). Therefore, the L-arginine-associated BaeS/BaeR-SalX pathway identified in this study is likely to function within a more complex intestinal ecosystem in which both host and microbial metabolism contribute to environmental signal availability.

These observations expand the growing repertoire of host-derived metabolites that modulate bacterial behavior and suggest that nutrient-associated signal integration constitutes an important strategy by which *V. cholerae* adapts to the intestinal environment. However, the present study does not establish whether L-arginine directly interacts with BaeS or activates the pathway indirectly through physiological changes in the bacterial cell. Further biochemical characterization will be required to define the molecular basis of BaeS/BaeR activation under L-arginine-associated conditions. In addition, we did not evaluate structurally related amino acids, including D-arginine and lysine, as potential activators of the BaeS/BaeR pathway. Consequently, although L-arginine was identified as a physiologically upstream cue under the experimental conditions tested, the ligand specificity of the BaeS/BaeR signaling system remains unresolved and will require systematic investigation in future studies.

Collectively, our findings position the BaeS/BaeR–SalX module as a signal-integration circuit that links host metabolic sensing to antimicrobial defense. Unlike previously characterized TCSs that primarily respond to envelope stress or antimicrobial molecules, this pathway couples responsiveness to L-arginine-associated conditions with activation of an antimicrobial peptide resistance effector, thereby providing a previously unrecognized mechanism by which *V. cholerae* coordinates metabolic adaptation, immune evasion, and colonization fitness during intestinal infection.

## Conclusion

In summary, our study establishes the BaeS/BaeR two-component system as a host metabolite-responsive regulatory hub that integrates L-arginine-associated pathway activation with antimicrobial peptide resistance and colonization control in *V. cholerae*. We define a functional L-arginine-associated BaeS/BaeR-SalX signaling axis in which phosphorylation-dependent activation of BaeR drives transcription of the ABC transporter gene *salX*, thereby enhancing bacterial adaptation to host-derived antimicrobial stress. Beyond identifying a single downstream effector, our transcriptomic analyses reveal that BaeS/BaeR orchestrates a broader adaptive regulon encompassing transport, metabolic, and stress-response pathways, underscoring its role as a global regulator of environmental fitness during infection. This regulatory architecture highlights a conserved strategy whereby two-component systems function as signal-integration modules coupling metabolite-associated pathway activation to defense effector expression. Collectively, these findings expand our understanding of how enteric pathogens integrate host metabolic cues into colonization regulation and define a functional model linking nutrient-associated signaling to antimicrobial resistance through a TCS-controlled effector system, without claiming direct L-arginine sensing by BaeS.

## Data Availability

The RNA-seq data generated in this study have been deposited in the NCBI Sequence Read Archive (SRA) under BioProject accession number PRJNA1422066 (https://www.ncbi.nlm.nih.gov/sra/PRJNA1422066).
