# Supplementary material for: The BaeS/BaeR two-component system enhances Vibrio cholerae intestinal colonization by upregulating salX
Source: Front Cell Infect Microbiol. 2026 Jul 23;16:1886586. doi: 10.3389/fcimb.2026.1886586 (PMC13442452; doi:10.3389/fcimb.2026.1886586)
Supplement: Supplementary file 1 [file Supplementaryfile1.docx]

**Supporting Information**

**The BaeS/BaeR two-component system enhances *Vibrio cholerae* intestinal colonization by upregulating *salX***

Qiushi Wang^1^, Xiaoya Guo^1^, Yongquan Shi^2^, Linyuan Xue^1^, Dongming Xing^1*^, Ting Ye^1*^

^1^Molecular Medicine Innovation Center, Cancer Institute of The Affiliated Hospital of Qingdao University, Qingdao University, Qingdao, China

^2^ Department of Clinical Laboratory Center, Shandong Second Provincial General Hospital, Jinan, China

***Corresponding author:** Ting Ye and Dongming Xing

Primary corresponding author. Ting Ye:1120220712@mail.nankai.edu.cn

Secondary corresponding author. Dongming Xing: xdm_tsinghua@163.com

**Figure S1**

**Figure S1.** Growth curves of WT, Δ*baeS/baeR* and Δ*baeS/baeR+* strains under LB medium.

**Figure S2**

**Figure S2****.** Relative *salX* transcript levels in WT and Δ*baeS/baeR* strains **(A)** grown in LB medium, **(B)** following 4 h of adhesion to Caco-2 cells, and **(C)** recovered from the small intestines of infected neonatal mice. Data are presented as mean ± SD from three biological replicates. Differences between groups were evaluated using an unpaired Student’s *t*-test. Exact *P* values are shown; n.s., not significant.

**Figure S3**

**Figure S3.** Growth of WT, Δ*salX,* Δ*baeR/salX* and Δ*baeR/salX+* strains in medium over 18 hours by OD_600_ readings.

**Figure S4**

**Figure S4.** **(A)** Survival of *V. cholerae* strains after exposure to 50 μg/mL HD-5 for 4 h (*n* = 3). **(B)** Minimum inhibitory concentrations (MICs) of HD-5 against WT, Δ*baeS/baeR*, Δ*salX*, Δ*baeR/salX*, Δ*baeS/baeR*+, and Δ*baeR/salX*+ strains (*n* = 3). Data are presented as mean ± SD from three independent biological replicates. Statistical significance was evaluated using an unpaired Student’s *t*-test; n.s., not significant.

**Figure S5**

**Figure S5. (A)** Western blot and **(B)** quantitative analysis of SalX protein abundance in WT and *baeR*-D91A strains cultured in LB medium. Data are shown as mean ± SD from three independent biological replicates. Statistical significance was evaluated using an unpaired Student’s *t*-test.

**Figure S6**

**Figure S6.** Intestinal luminal L-arginine concentrations in adult and neonatal mice. Each point represents one mouse (*n* = 6 per group). Data are presented as mean ± SD. Statistical significance was evaluated using an unpaired Student’s *t*-test.

**Figure S7**

**Figure S7.** qRT-PCR analysis of *salX* expression in WT, *baeS*-H410A, and *baeR*-D91A strains grown in M9 minimal medium supplemented with 20, 50, 100, or 150 μM L-arginine (*n* = 3). Data are shown as mean ± SD from three independent biological replicates. Statistical significance was evaluated using two-way ANOVA; n.s., not significant.

**Figure S8**

**Figure S8**. EMSA analysis of BaeR binding to *salX* promoter deletion fragments. **(A)** EMSA using the P*_salX_*-2 fragment, in which 5′-CCGATAGT-3′ was deleted from the −10/−35 spacer region. **(B)** EMSA using the P*_salX_*-3 fragment, in which 5′-TTCTTTTT-3′ was deleted from the −10/−35 spacer region.

**Figure S9**

**Figure S9.** Kaplan–Meier survival curves of neonatal mice orally infected with WT, Δ*baeS/baeR*, Δ*salX*, Δ*baeR/salX*, Δ*baeS/baeR*+, or Δ*baeR/salX*+ strains. Each group contained six mice. Statistical significance was determined using the log-rank (Mantel–Cox) test. n.s., not significant.
